# Nursing in the Brazilian prison system: between the right to health and the challenges of custody

**DOI:** 10.1590/1518-8345.0000.5126

**Published:** 2026-10-09

**Authors:** Roberto Dias de Oliveira, Andrea da Silva Santos, Thiago Nascimento do Prado

**Affiliations:** 1 Universidade Estadual de Mato Grosso do Sul, Curso de Enfermagem, Dourados, MS, Brazil. roberto@uems.br; 2 Universidade Federal da Grande Dourados, Faculdade de Ciências da Saúde, Dourados, MS, Brazil.; 3 Universidade Federal do Espírito Santo, Departamento de Enfermagem, Vitória, ES, Brazil.

**Figure:**
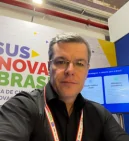

**Figure:**
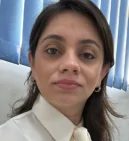

**Figure:**
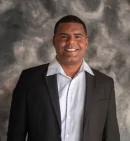

In the Latin American prison context, historically marked by chronic overcrowding, poor sanitary conditions, and persistent human rights violations, nursing plays a central and often solitary role in guaranteeing access to health for people deprived of liberty. In Brazil - a country that currently holds the third largest prison population in the world -, this professional performance is simultaneously indispensable for the maintenance of life and severely limited by structural, organizational, and security barriers. This editorial proposes a reflection on the complex challenges that permeate this practice, arguing that strengthening prison nursing is a fundamental (and urgent) condition to transform the constitutional right to health into effective, comprehensive, humanized care that promotes citizenship.

Health is an inalienable fundamental right, guaranteed by the Brazilian Constitution and supported by the principles of the Unified Health System (SUS). However, when looking at the population deprived of liberty (PPL), the fulfillment of this right is crossed by deep inequalities, being constantly permeated by the tension between the mandatory demands of security and the essence of care^1^. In this context of deprivation of liberty, the care practice of the nurse constitutes, in the overwhelming majority of cases, the main - if not the only - link between the custody individual and the public health system.

Due to the scarcity of resources and the high demand for illnesses in prison, this performance becomes predominantly curative and focused on the complaint-conduct model. Literature indicates that the preparation, continuous administration of medication, and immediate clinical follow-up occupy most of the nursing workload^2^. In contrast, strategic actions of health education, harm prevention, and well-being promotion receive low institutional priority. This predominance of a biomedical and reactive model is not a deliberate choice of the professional, but rather a reflection of the adverse conditions imposed by the institutions, highlighting the urgency of rethinking staff sizing and reorganizing the work process in penal units.

Beyond strictly operational challenges, nursing practice in prison is permeated by ethical and identity tensions, as well as complex power relations. The organizational culture of prisons is historically oriented by punitive logic, surveillance, and rigorous discipline. This institutional architecture tends to subordinate technical and scientific health knowledge to the strict requirements of custody^3^ ^), (^ ^4^. As a result, the nurse is inserted into a constant dilemma, often described as a conflict of “dual loyalty”: they must balance their deontological commitment to human rights and the relief of suffering against institutional rules that prioritize security in a hostile and frequently stigmatized environment.

The physical precariousness of the units, combined with the scarcity of basic supplies and the frequent absence of multidisciplinary teams, restricts the nurse’s autonomy. This scenario generates invisibility, low professional recognition, and imposes constant moral dilemmas in daily decision-making^3^ ^), (^ ^4^ ^), (^ ^5^. Despite this largely restrictive reality, the ideal scope of nursing practice requires the development of highly complex clinical care, adapted to the extreme social, sanitary, and epidemiological vulnerability typical of prisons - environments conducive to the spread of infectious and parasitic diseases and the worsening of mental disorders.

In their expanded role, health surveillance actions and humanized care with particularly vulnerable groups must stand out, such as sexual and reproductive health care for women deprived of liberty. This practice, therefore, requires high technical, relational, and emotional competencies from the nurse to act in a territory of permanent tension, in which the quality and confidentiality of care frequently compete for space with searches, counts, and security routines.

To overcome this scenario of invisibility and precarization, structural, political, and educational changes are urgent. First, the need to implement a specific curricular training in forensic nursing and prison health stands out, both in undergraduate courses and in residency and postgraduate programs. Second, the development and application of intersectoral clinical protocols that articulate guidelines from the health and justice sectors are fundamental, backing the nurse’s decision-making and protecting the act of care^1^ ^), (^ ^3^.

Allied to this, strategic planning of human and material resources, as well as massive investment in intramural health infrastructure, are indispensable to guarantee the quality and continuity of care^2^ ^), (^ ^4^. The nurse, properly supported by valuation policies and a specific career path, must possess the conditions and autonomy to lead the management of prison health services, epidemiological surveillance, permanent education of their team, and the implementation of practices based on solid scientific evidence.

The performance of the nurse in the Brazilian prison system is, ultimately, the materialization of social justice at the frontline of the system. Discussing nursing in this context means recognizing that the real effectiveness of a country’s public health policies is also measured by the State’s capacity to care for its historically most invisible and marginalized populations.

Strengthening specialized training, guaranteeing technical autonomy, providing decent working conditions, and ensuring institutional support do not represent merely class demands of nursing; these are ethical commitments intrinsic to the defense of human rights^5^. The scientific community, assistance nurses, managers, and public policy makers face the great challenge of definitively placing prison health at the center of the academic and governmental agenda. Only under this premise will nursing fully fulfill its social vocation to care, protect, and reaffirm the non-negotiable dignity of all people, regardless of their condition of freedom.

## Declaration on the use of Artificial Intelligence resources

We declare that, for the literature review of this editorial, the artificial intelligence tool Consensus (https://consensus.app) was used, accessed on July 15, 2026. The tool was employed exclusively for the identification, screening, and summarization of published evidence related to the topic of the editorial. All results generated by the AI were critically evaluated by the authors. The authors assume full responsibility for the accuracy, integrity, and originality of the content, confirming that there is no plagiarism in the parts of the manuscript influenced by the AI and that all sources were properly cited.
