# Supplementary material for: Nursing in the Brazilian prison system: between the right to health and the challenges of custody
Source: Rev Lat Am Enfermagem. 2026 Oct 9;34:e5126. doi: 10.1590/1518-8345.0000.5126 (PMC13652532; doi:10.1590/1518-8345.0000.5126)
Supplement: Translation

# Translation: Enfermagem no sistema prisional brasileiro: entre o direito à saúde e os desafios da custódia

**DOI:** 10.1590/1518-8345.0000.5127

**Authors:** Roberto Dias de Oliveira, Andrea da Silva Santos, Thiago Nascimento do Prado

**Affiliations:** 1 Universidade Estadual de Mato Grosso do Sul, Curso de Enfermagem, Dourados, MS, Brasil. roberto@uems.br; 2 Universidade Federal da Grande Dourados, Faculdade de Ciências da Saúde, Dourados, MS, Brasil.; 3 Universidade Federal do Espírito Santo, Departamento de Enfermagem, Vitória, ES, Brasil.

No cenário prisional latino-americano, historicamente marcado por superlotação crônica, precariedade sanitária e violações persistentes de direitos humanos, a enfermagem desempenha um papel central e, muitas vezes, solitário na garantia do acesso à saúde das pessoas privadas de liberdade. No Brasil - país que atualmente abriga a terceira maior população carcerária do mundo -, essa atuação profissional mostra-se simultaneamente indispensável para a manutenção da vida e severamente limitada por barreiras estruturais, organizacionais e de segurança. Este editorial propõe uma reflexão sobre os desafios complexos que permeiam essa prática, defendendo que o fortalecimento da enfermagem prisional é uma condição fundamental (e inadiável) para transformar o direito constitucional à saúde em um cuidado efetivo, integral, humanizado e promotor de cidadania.

A saúde é um direito fundamental inalienável, assegurado pela Constituição Brasileira e amparado pelos princípios do Sistema Único de Saúde (SUS). Entretanto, quando o olhar se volta para a população privada de liberdade (PPL), a efetivação desse direito é atravessada por profundas desigualdades, sendo constantemente permeada pela tensão entre as demandas impositivas de segurança e a essência do cuidado^1^. Neste contexto de privação de liberdade, a prática assistencial do enfermeiro constitui, na esmagadora maioria das vezes, o principal - senão o único - elo entre o indivíduo custodiado e o sistema de saúde público.

Devido à escassez de recursos e à alta demanda de adoecimento no cárcere, essa atuação torna-se predominantemente curativa e focada na queixa-conduta. A literatura aponta que o preparo, a administração contínua de medicamentos e o acompanhamento clínico imediato ocupam a maior parte do tempo de trabalho da enfermagem^2^. Em contrapartida, as ações estratégicas de educação em saúde, a prevenção de agravos e a promoção do bem-estar recebem baixa prioridade institucional. Essa predominância de um modelo biomédico e reativo não é uma escolha deliberada do profissional, mas sim o reflexo das condições adversas impostas pelas instituições, evidenciando a urgência de se repensar o dimensionamento de pessoal e reorganizar o processo de trabalho nas unidades penais.

Para além dos desafios estritamente operacionais, a prática da enfermagem no cárcere é permeada por tensões éticas, identitárias e por complexas relações de poder. A cultura organizacional das prisões é historicamente orientada pela lógica punitiva, pela vigilância e pela disciplina rigorosa. Essa arquitetura institucional tende a subordinar o saber técnico e científico da saúde às exigências estritas da custódia^3^ ^), (^ ^4^. Com isso, o enfermeiro se vê inserido em um dilema constante, muitas vezes descrito como um conflito de “dupla lealdade”: deve equilibrar o seu compromisso deontológico com os direitos humanos e o alívio do sofrimento diante de regras institucionais que priorizam a segurança em um ambiente hostil e frequentemente estigmatizado.

A precariedade física das unidades, somada à escassez de insumos básicos e à ausência frequente de equipes multiprofissionais, restringe a autonomia do enfermeiro. Esse cenário gera invisibilidade, baixo reconhecimento profissional e impõe constantes dilemas morais na tomada de decisão diária^3^ ^), (^ ^4^ ^), (^ ^5^. Apesar dessa realidade amplamente restritiva, o escopo ideal de atuação da enfermagem exige o desenvolvimento de um cuidado clínico altamente complexo, adaptado à extrema vulnerabilidade social, sanitária e epidemiológica típica das prisões - ambientes propícios à disseminação de doenças infecciosas e parasitárias e ao agravamento de transtornos mentais.

Em seu papel ampliado, devem destacar-se as ações de vigilância em saúde e o cuidado humanizado com grupos particularmente vulnerabilizados, a exemplo da atenção à saúde sexual e reprodutiva das mulheres privadas de liberdade. Essa prática, portanto, exige do enfermeiro altas competências técnicas, relacionais e emocionais para atuar em um território de tensão permanente, no qual a qualidade e o sigilo do cuidado frequentemente disputam espaço com as revistas, contagens e rotinas de segurança.

Para superar esse cenário de invisibilidade e precarização, são urgentes mudanças de ordem estrutural, política e educacional. Em primeiro lugar, destaca-se a necessidade de implantação de uma formação curricular específica em enfermagem forense e saúde prisional, tanto nos cursos de graduação quanto nos programas de residência e pós-graduação. Em segundo lugar, são fundamentais a elaboração e a aplicação de protocolos clínicos intersetoriais, que articulem as diretrizes das pastas da Saúde e da Justiça, respaldando a tomada de decisão do enfermeiro e protegendo o ato de cuidar^1^ ^), (^ ^3^.

Aliado a isso, o planejamento estratégico de recursos humanos e materiais, bem como o investimento maciço em infraestrutura de saúde intramuros, são indispensáveis para garantir a qualidade e a continuidade da assistência^2^ ^), (^ ^4^. O enfermeiro, devidamente amparado por políticas de valorização e por uma carreira específica, deve reunir condições e autonomia para liderar a gestão dos serviços de saúde prisionais, a vigilância epidemiológica, a educação permanente de sua equipe e a implementação de práticas baseadas em evidências científicas sólidas.

A atuação do enfermeiro no sistema prisional brasileiro é, em última análise, a materialização da justiça social na ponta do sistema. Discutir a enfermagem neste contexto significa reconhecer que a real efetividade das políticas públicas de saúde de um país também se mede pela capacidade do Estado de cuidar de suas populações historicamente mais invisibilizadas e marginalizadas.

Fortalecer a formação especializada, garantir a autonomia técnica, prover condições dignas de trabalho e assegurar o apoio institucional não representam apenas demandas classistas da enfermagem; tratam-se de compromissos éticos intrínsecos à defesa dos direitos humanos^5^. À comunidade científica, aos enfermeiros assistenciais, gestores e formuladores de políticas públicas cabe o grande desafio de alocar a saúde prisional, definitivamente, no centro da agenda acadêmica e governamental. Somente sob essa premissa a enfermagem cumprirá plenamente sua vocação social de cuidar, proteger e reafirmar a dignidade inegociável de todas as pessoas, independentemente de sua condição de liberdade.

## Declaração sobre o uso de recursos de Inteligência Artificial

Declaramos que, para a revisão bibliográfica deste editorial, foi utilizada a ferramenta de inteligência artificial Consensus (https://consensus.app), acessada em 15 de julho de 2026. A ferramenta foi empregada exclusivamente para identificação, triagem e sumarização de evidências publicadas relacionadas ao tema do editorial. Todos os resultados gerados pela IA foram criticamente avaliados pelos autores. Os autores assumem plena responsabilidade pela precisão, integridade e originalidade do conteúdo, confirmando que não há plágio nas partes do manuscrito influenciadas pela IA e que todas as fontes foram devidamente citadas.

# Translation: Enfermería en el sistema penitenciario brasileño: entre el derecho a la salud y los desafíos de la custodia

**DOI:** 10.1590/1518-8345.0000.5125

**Authors:** Roberto Dias de Oliveira, Andrea da Silva Santos, Thiago Nascimento do Prado

**Affiliations:** 1 Universidade Estadual de Mato Grosso do Sul, Curso de Enfermagem, Dourados, MS, Brasil. roberto@uems.br; 2 Universidade Federal da Grande Dourados, Faculdade de Ciências da Saúde, Dourados, MS, Brasil.; 3 Universidade Federal do Espírito Santo, Departamento de Enfermagem, Vitória, ES, Brasil.

In el escenario penitenciario latinoamericano, históricamente marcado por el hacinamiento crónico, la precariedad sanitaria y las violaciones persistentes de los derechos humanos, la enfermería desempeña un papel central y, a menudo, solitario en la garantía del acceso a la salud de las personas privadas de libertad. En Brasil - país que actualmente alberga a la tercera mayor población carcelaria del mundo -, esta actuación profesional se muestra simultáneamente indispensable para la manutención de la vida y severamente limitada por barreras estructurales, organizativas y de seguridad. Este editorial propone una reflexión sobre los complejos desafíos que permean esta práctica, defendiendo que el fortalecimiento de la enfermería penitenciaria es una condición fundamental (e inaplazable) para transformar el derecho constitucional a la salud en un cuidado efectivo, integral, humanizado y promotor de ciudadanía.

La salud es un derecho fundamental inalienable, asegurado por la Constitución Brasileña y amparado por los principios del Sistema Único de Salud (SUS). Sin embargo, cuando la mirada se vuelve hacia la población privada de libertad (PPL), la efectivización de este derecho es atravesada por profundas desigualdades, estando constantemente permeada por la tensión entre las demandas impositivas de seguridad y la esencia del cuidado^1^. En este contexto de privación de libertad, la práctica asistencial del enfermero constituye, en la abrumadora mayoría de las veces, el principal - sino el único - vínculo entre el individuo custodiado y el sistema de salud público.

Debido a la escasez de recursos y a la alta demanda de morbilidad en el cárcel, esta actuación se vuelve predominantemente curativa y enfocada en el modelo de queja-conducta. La literatura señala que la preparación, la administración continua de medicamentos y el seguimiento clínico inmediato ocupan la mayor parte del tiempo de trabajo de la enfermería^2^. En contrapartida, las acciones estratégicas de educación en salud, la prevención de riesgos y la promoción del bienestar reciben una baja prioridad institucional. Esta predominancia de un modelo biomédico y reactivo no es una elección deliberada del profesional, sino el reflejo de las condiciones adversas impuestas por las instituciones, evidenciando la urgencia de replantear el dimensionamiento del personal y reorganizar el proceso de trabajo en las unidades penales.

Más allá de los desafíos estrictamente operativos, la práctica de la enfermería en el cárcel está permeada por tensiones éticas, identitarias y por complejas relaciones de poder. La cultura organizacional de las prisiones está históricamente orientada por la lógica punitiva, la vigilancia y la disciplina rigurosa. Esta arquitectura institucional tiende a subordinar el saber técnico y científico de la salud a las exigencias estrictas de la custodia^3^ ^), (^ ^4^. Con ello, el enfermero se ve inserto en un dilema constante, a menudo descrito como un conflicto de “doble lealtad”: debe equilibrar su compromiso deontológico con los derechos humanos y el alivio del sufrimiento frente a reglas institucionales que priorizan la seguridad en un entorno hostil y frecuentemente estigmatizado.

La precariedad física de las unidades, sumada a la escasez de insumos básicos y a la ausencia frecuente de equipos multiprofesionales, restringe la autonomía del enfermero. Este escenario genera invisibilidad, bajo reconocimiento profesional e impone constantes dilemas morales en la toma de decisiones diaria^3^ ^), (^ ^4^ ^), (^ ^5^. A pesar de esta realidad ampliamente restrictiva, el alcance ideal de la actuación de la enfermería exige el desarrollo de un cuidado clínico altamente complejo, adaptado a la extrema vulnerabilidad social, sanitaria y epidemiológica típica de las prisiones - ambientes propicios para la diseminación de enfermedades infecciosas y parasitarias y para el agravamiento de los trastornos mentales.

En su rol ampliado, deben destacarse las acciones de vigilancia en salud y el cuidado humanizado con grupos particularmente vulnerabilizados, a ejemplo de la atención a la salud sexual y reproductiva de las mujeres privadas de libertad. Esta práctica, por lo tanto, exige del enfermero altas competencias técnicas, relacionales y emocionales para actuar en un territorio de tensión permanente, en el cual la calidad y el secreto del cuidado frecuentemente disputan espacio con las requisas, los recuentos y las rutinas de seguridad.

Para superar este escenario de invisibilidad y precarización, son urgentes cambios de orden estructural, político y educativo. En primer lugar, se destaca la necesidad de implantación de una formación curricular específica en enfermería forense y salud penitenciaria, tanto en los cursos de grado como en los programas de residencia y posgrado. En segundo lugar, son fundamentales la elaboración y la aplicación de protocolos clínicos intersectoriales, que articulen las directrices de las carteras de Salud y de Justicia, respaldando la toma de decisiones del enfermero y protegiendo el acto de cuidar^1^ ^), (^ ^3^.

Aliado a esto, el planeamiento estratégico de recursos humanos y materiales, así como la inversión masiva en infraestructura de salud intramuros, son indispensables para garantizar la calidad y la continuidad de la asistencia^2^ ^), (^ ^4^. El enfermero, debidamente amparado por políticas de valorización y por una carrera específica, debe reunir condiciones y autonomía para liderar la gestión de los servicios de salud penitenciarios, la vigilancia epidemiológica, la educación permanente de su equipo y la implementación de prácticas basadas en evidencias científicas sólidas.

La actuación del enfermero en el sistema penitenciario brasileño es, en última instancia, la materialización de la justicia social en la primera línea del sistema. Discutir la enfermería en este contexto significa reconocer que la real efectividad de las políticas públicas de salud de un país también se mide por la capacidad del Estado de cuidar a sus poblaciones históricamente más invisibilizadas y marginadas.

Fortalecer la formación especializada, garantizar la autonomía técnica, proveer condiciones dignas de trabajo y asegurar el apoyo institucional no representan solo demandas clasistas de la enfermería; se trata de compromisos éticos intrínsecos a la defensa de los derechos humanos^5^. A la comunidad científica, a los enfermeros asistenciales, gestores y formuladores de políticas públicas les corresponde el gran desafío de ubicar la salud penitenciaria, definitivamente, en el centro de la agenda académica y gubernamental. Solo bajo esta premisa la enfermería cumplirá plenamente su vocación social de cuidar, proteger y reafirmar la dignidad negociable de todas las personas, independientemente de su condición de libertad.

## Declaración sobre el uso de recursos de Inteligencia Artificial

Declaramos que, para la revisión bibliográfica de este editorial, se utilizó la herramienta de inteligencia artificial Consensus (https://consensus.app), accedida el 15 de julio de 2026. La herramienta fue empleada exclusivamente para la identificación, cribado y resumen de evidencias publicadas relacionadas con el tema del editorial. Todos los resultados generados por la IA fueron evaluados críticamente por los autores. Los autores asumen plena responsabilidad por la precisión, integridad y originalidad del contenido, confirmando que no existe plagio en las partes del manuscrito influenciadas por la IA y que todas las fuentes fueron debidamente citadas.
